# Dual application of duckweed and azolla plants for wastewater treatment and renewable fuels and petrochemicals production

**DOI:** 10.1186/1754-6834-7-30

**Published:** 2014-02-28

**Authors:** Nazim Muradov, Mohamed Taha, Ana F Miranda, Krishna Kadali, Amit Gujar, Simone Rochfort, Trevor Stevenson, Andrew S Ball, Aidyn Mouradov

**Affiliations:** 1 University of Central Florida, Florida Solar Energy Centre, 1679 Clearlake Road, 32922 Cocoa, FL, USA; 2 Royal Melbourne Institute of Technology University, 3083 Bundoora, VIC, Australia; 3 Department of Primary Industries, Biosciences Research Division, Victorian AgriBiosciences Centre, 3083 Bundoora, Victoria, Australia

**Keywords:** Algae, Biofuel, Bioremediation, Pyrolysis, Swine wastewater, Thermochemical conversion

## Abstract

**Background:**

Shortages in fresh water supplies today affects more than 1 billion people worldwide. Phytoremediation strategies, based on the abilities of aquatic plants to recycle nutrients offer an attractive solution for the bioremediation of water pollution and represents one of the most globally researched issues. The subsequent application of the biomass from the remediation for the production of fuels and petrochemicals offers an ecologically friendly and cost-effective solution for water pollution problems and production of value-added products.

**Results:**

In this paper, the feasibility of the dual application of duckweed and azolla aquatic plants for wastewater treatment and production of renewable fuels and petrochemicals is explored. The differences in absorption rates of the key wastewater nutrients, ammonium and phosphorus by these aquatic macrophytes were used as the basis for optimization of the composition of wastewater effluents. Analysis of pyrolysis products showed that azolla and algae produce a similar range of bio-oils that contain a large spectrum of petrochemicals including straight-chain C10-C21 alkanes, which can be directly used as diesel fuel supplement, or a glycerin-free component of biodiesel. Pyrolysis of duckweed produces a different range of bio-oil components that can potentially be used for the production of “green” gasoline and diesel fuel using existing techniques, such as catalytic hydrodeoxygenation.

**Conclusions:**

Differences in absorption rates of the key wastewater nutrients, ammonium and phosphorus by different aquatic macrophytes can be used for optimization of composition of wastewater effluents. The generated data suggest that the composition of the petrochemicals can be modified in a targeted fashion, not only by using different species, but also by changing the source plants’ metabolic profile, by exposing them to different abiotic or biotic stresses. This study presents an attractive, ecologically friendly and cost-effective solution for efficient bio-filtration of swine wastewater and petrochemicals production from generated biomass.

## Background

Aquatic macrophytes improve water quality by accumulating toxic nutrients and heavy metals and by regulating oxygen balance. Various aquatic floating macrophytes have been proposed as agents of choice for the bioremediation of wastewater because of these features and also their very high growth rates and easy maintenance. Among the macrophytes most used for bioremediation are representatives of *Lemnaceae* or duckweeds (*Lemna* spp., *Spirodela spp*., *Wolffia* spp and *Wolffiella* spp); *Eichhornia crassipes*, *Hydrocotyle umbellata* and water fern azolla (*Azolla filiculoides* (*A. filiculoides*)) [1-8]. Duckweed species have been used for over 30 years to recover nutrients from wastewaters [9-18]. They are also a good source of proteins and starch, which are essential for the production of value-added products such as animal feed and also for bio-ethanol applications [19-21].

Azolla, a free-floating water fern is another fast-growing plant, which can grow in the absence of nitrogen (N) in water because of a symbiotic association with the nitrogen-fixing endophytic blue-green cyanobacterium, *Anabaena azollae* Strasburger (*A. azollae*), shown in Additional file 1. This endosymbiont provides sufficient nitrogen for both itself and its host and is using the fern as a protected environment and source of carbohydrates [22]. The rate of nitrogen fixation of *A. azollae* is estimated at 1.1 t N/ha-year, which is significantly higher than the nitrogen fixation rate of the legumes, 0.4 t N/ha-year [23]. Azolla species have been intensively studied due to their high growth rates combined with high bioremediation efficiency [1,3,5-7,24-26]. Similarly to duckweed, azolla biomass has been used as an animal feed. Azolla is rich in proteins, essential amino acids, vitamins A, B12, beta-carotene, growth promoter intermediates and minerals like calcium, phosphorus, potassium, iron, copper and magnesium. These nutrient values, together with the low lignin content makes them a useful nutrient feed for livestock. Being rich in phosphorus (P) and N, azolla has been successfully used as a green manure in rice fields in Asia and Africa and as a feed supplement for aquatic and terrestrial animals [1,2].

The exploitation of aquatic macrophytes as feedstock for biofuel production has so far focused on the saccharification of cell walls for the production of reduced sugars for conversion to value-added products or ethanol [20,27,28]. In the case of duckweed, relatively high level of starch, a favorable composition of cell-wall carbohydrates and lack or low level of lignin can reduce the pretreatment and enzyme dosages, significantly improving the economics of bio-ethanol production. As a result, the theoretical ethanol yield of duckweed can reach 6.42 × 10^3^ L ha^-1^, about 50% higher than that of maize-based ethanol production, which makes duckweed a competitive starch source for fuel ethanol production [21].

Pyrolysis, the thermal decomposition (400 to 550°C) of organic compounds in the absence of air/oxygen has significant advantages over other bio- and thermochemical methods. The pyrolysis process produces valuable products, such as pyrolysis gas, bio-oil and bio-solids (char and mineral ash), which may be directly used as fuels or represent feedstocks for the petrochemical industry. The fact that the toxic contaminants, such as heavy metals accumulated in feedstock can be safely enclosed in the solid fractions such as a char and/or ash makes pyrolysis a technology of choice for conversion of waste biomass into the value-added products. Most of the pyrolysis studies so far have been carried out with cellulosic feedstocks, which are mainly composed of cellulose, hemicelluloses and lignin [29-31]. In spite of the impressive biomass accumulation in plants and the fact that the chemical composition of aquatic species consists mainly of carbohydrates, proteins and lipids which can also potentially be thermo-converted into bio-oil, only the thermal transformation of algal species into biofuel has been intensively studied [32-34]. Recently, we have optimized conditions for pyrolysis of *Lemna minor*, which showed a great potential for duckweed as feedstock for production of bio-oil and bio-char [35,36]. It has also been demonstrated that the crude bio-oil from the hydrothermal liquefaction of duckweed (*Lemna* sp.) can be effectively upgraded in subcritical water [37].

In this paper, we explore the possibility of the dual application of duckweed and azolla plants for efficient wastewater treatment and the production of renewable fuels and petrochemicals. The study aimed to assess phenological behaviour, phytomass production and chemical composition of two common Australian representatives of water-floating macrophytes: a duckweed, *Landoltia punctata* (*L. punctata*) and a water fern azolla, *A. filiculoides*. The assessment included production of renewable value products such as bio-gas, carbon-rich bio-solids and liquid petrochemicals from generated biomass collected after wastewater treatment. The detailed study of bioremediation efficiency and biochemical composition of feedstocks was linked with identification and quantitative analysis of the main pyrolysis products.

## Results and discussion

### Application of duckweed and azolla for swine wastewater treatment

#### Application of duckweed and azolla for swine wastewater treatment and algae removal

**Figure 1:**
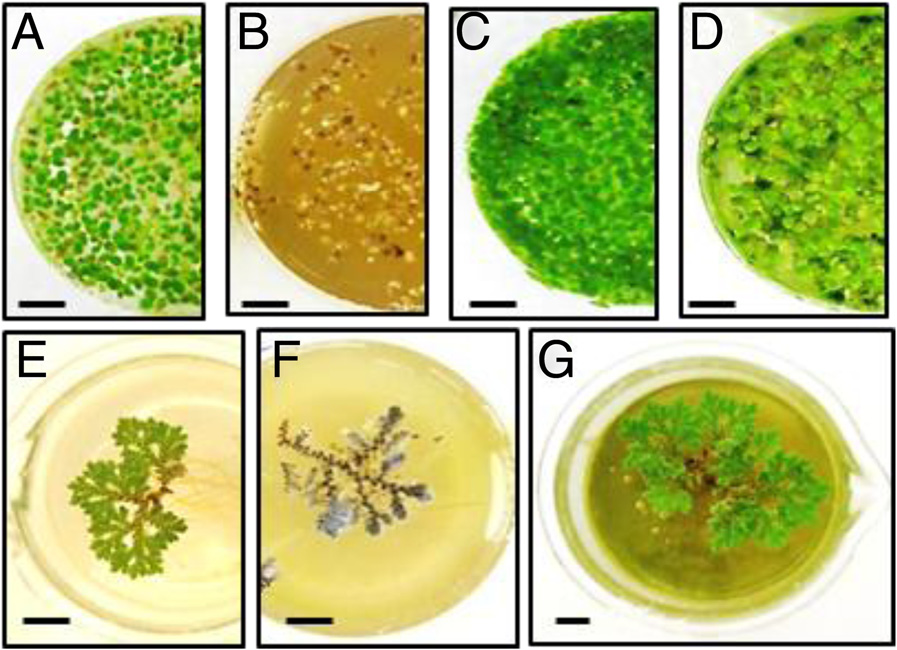
**Growth of duckweed and azolla in different dilutions of anaerobically digested swine wastewater (ADSW). (A-D)** Growing duckweed; **(E-G)** Growing azolla; **(A)** and **(E)** controls; **(B)** 50% ADSW; **(C)** 10% ADSW; **(D)** 2.5% ADSW; **(F)** 15% ADSW; **(G)** 2.5% ADSW; bar =1 cm.

The ability of duckweed and azolla cultures to grow and remediate nutrients (NH_4_^+^-N and PO_4_^-3^-P) from different dilutions of anaerobically digested swine wastewater (ADSW) prepared from swine lagoon wastewaters was assessed (Additional file 2, Table 1 and Table 2). ADSW showed inhibitory effects on duckweed growth down to 15% concentrations with damaged and necrotic fronds observed after 2 to 3 days of growth (Figure 1, Table 1). The duckweed grew well in 10 to 1.2% ADSW with the highest growth rate, 5.4 g dry weight (dw)/m^2^-day, observed in 10% ADSW. This growth rate was associated with an uptake rate of NH_4_^+^-N, 5.8 mg/L-day (87% uptake), and of PO_4_^-3^-P, 0.7 mg/L-day (45% uptake). The rate of nutrient uptake was lower than that observed [16,17] for *Spirodela punctata* (22 mg/L-day) grown both under lab conditions and in an outdoor field test over 60 days using anaerobically digested swine wastewater (2.03 g/m^2^-day of total Kjehldahl nitrogen (TKN) and 0.4 g/m^2^-day for total phosphorus (TP) ) [16,38]. Our data, however, show good agreement, for both nutrient removal and specific growth rate, with those for S*pirodela. oligorrhiza* in 6% swine lagoon water concentration (3.5 mg N/L-day and 0.35 mg P/L-day) [20], and are higher than N and P levels found previously [39] (0.025 mg/L-day) after growing in storm wastewater.

**Table 1:** Growth rates of duckweed, nutrients removals and chlorophyll concentrations in different dilutions of ADSW

**Duckweed *(L. punctata)***									
**ADSW dilutions**	**Growth rate, g dw/m2-day**	**NH4-N, mg/L, Final**	**NH4-N uptake, %**	**NH4-N uptake rate, mg/l-day**	**PO4-P, mg/L, Final**	**PO4-P uptake, %**	**PO4-P uptake rate, mg/L-day**	**N/P uptake**	**Chl A + B μg/ml**
ADSW, 100%	NA	NA	NA	NA	NA	NA	NA	NA	NA
ADSW, 50%	NA	NA	NA	NA	NA	NA	NA	NA	NA
ADSW, 25%	-1.6	105.1 ± 11.4	36.0 ± 3.9	5.92 ± 1.7	37.1 ± 3.3	4.1 ± 1.8	0.16 ± 0.07	NA	5.9 ± 1.8
ADSW, 15%	-1.2	45.9 ± 3.3	54.9 ± 5.5	5.5 ± 1.2	21.9 ± 3.0	11.3 ± 2.8	0.28 ± 0.08	NA	6.1 ± 1.1
ADSW, 10%	5.4 ± 2.3	8.4 ± 1.7	87.3 ± 6.5	5.8 ± 1.9	8.8 ± 3.7	45.3 ± 3.0	0.73 ± 0.2	7.90	5.1 ± 2.4
ADSW, 5%	4.9 ± 2.2	2.11 ± 1.1	93.5 ± 9.3	3.0 ± 1.1	4.3 ± 3.0	46.9 ± 4.9	0.38 ± 0.07	7.69	4.6 ± 2.2
ADSW, 2.5%	4.3 ± 1.7	0.0	100.0	1.6 ± 0.7	2.1 ± 1.7	54.3 ± 3.7	0.25 ± 0.09	6.44	3.1 ± 1.0
ADSW, 1.2%	3.0 ± 1.6	0.0	100.0	0.88 ± 0.3	1.0 ± 0.8	60.1 ± 4.8	0.15 ± 0.04	5.83	2.1 ± 0.1

ADSW, anaerobically digested swine wastewater; N, nitrogen; P, phosphorus; NA (not analyzed).

**Table 2:** Growth rates of azolla, associated nutrients removals and chlorophyll concentrations in different dilutions of ADSW

**Azolla *(A. filiculoides)***									
**ADSW dilutions**	**Growth rate, g dw/m2-day**	**NH4-N, mg/L, Final**	**NH4-N uptake,%**	**NH4-N uptake rate, mg/l-day**	**PO4-P, mg/L, Final**	**PO4-P uptake,%**	**PO4-P uptake rate, mg/l-day**	**N/P uptake**	**Chl A + B μg/ml**
ADSW, 100%	NA	NA	NA	NA	NA	NA	NA	NA	NA
ADSW, 50%	NA	NA	NA	NA	NA	NA	NA	NA	NA
ADSW, 25%	NA	NA	NA	NA	NA	NA	NA	NA	NA
ADSW, 15%	-1.5	58 ± 5.7	43.0 ± 3.3	4.38 ± 1.9	23.5 ± 3.9	4.8 ± 1.1	0.12 ± 0.07	NA	6.1 ± 1.1
ADSW, 10%	-1.3	28.8 ± 3.3	56.4 ± 3.8	3.73 ± 0.7	10.2 ± 2.2	36.6 ± 3.9	0.59 ± 0.3	6.32	4.1 ± 1.0
ADSW, 5%	1.1 ± 0.1	22.1 ± 2.5	31.2 ± 4.8	1.2 ± 0.3	6.4 ± 1.0	21.4 ± 4.7	0.17 ± 0.03	5.88	2.8 ± 0.8
ADSW, 2.5%	5.6 ± 1.8	0.0	100.0	1.61 ± 0.9	0.8 ± 0.07	82.6 ± 7.6	0.38 ± 0.07	4.30	1.9 ± 0.9
ADSW, 1.2%	4.6 ± 1.1	0.0	100.0	0.88 ± 0.1	0.0	100.0	0.25 ± 0.05	3.51	0.1 ± 0.08

ADSW, anaerobically digested swine wastewater; N, nitrogen; P, phosphorus; NA (not analyzed).

The reduction in efficiency of PO_4_^-3^-P uptake (up to 4.8-fold) in more diluted ADSW confirmed the requirement of high levels of N for efficient bioremediation of P, which has been reported in previous publications [20]. An average ratio of NH_4_^+^-N/PO_4_^-3^-P uptake was 6.7 which is in agreement with most published data, where this ratio ranges from 2.5 to 10.0, with the majority reported around 7.0 [10,17,20,38,40]. Reduction in concentrations of both nutrients led to minor decreases in the growth rates of duckweed, 5.4 to 4.3 g dw/m^2^-day for 10 to 2.5% ADSW, respectively, but this effect was diminished in 1.2% ADSW (3.3 g dw/m^2^-day), in agreement with an early finding [20].

Azolla plants showed higher sensitivity to ADSW, turning brown from the centre of the fronds and eventually dying after days 5 to 7 at concentrations of 50 to10% (Figure 1, Table 2). The highest azolla production was obtained in 2.5% ADSW; under these growth conditions azolla showed 100% absorption of NH_4_^+^-N and 83% uptake of PO_4_^-3^-P. The nitrogen uptake rate was similar to the rates observed previously [4] (155.9 to 252.5 mg N/m^2^–day, or around 3 mg/L-day), for *Azolla japonica*, grown in swine wastewater (2.5 mg/L-day) and for *Lemna gibba* grown in domestic wastewater (120 to 209 mg N/m^2^–day) [41]. This rate, however, is higher than those reported [42] (99 mg N/m^2^–day) and for azolla grown in fish farm wastewater, which is low in both nutrients [41], but lower than those reported by Vicenzini *et al*., [43] (370 to 540 mg N/m^2^–day), for azolla grown in outdoor culture. The ratio of NH_4_^+^-N/PO_4_^-3^-P absorption rates for azolla in our study was between 3.5 and 6.3, which is in agreement with other publications [4,44], but lower than that observed for duckweed (this research), probably because of higher rates of PO_4_^-3^-P uptake in solutions with low concentrations of NH_4_^+^-N by azolla. As a result the growth rates of azolla at low concentrations of ADSW (2.5% and 1.2%) were higher than those of duckweed. *A. japonica* showed a 4.5-fold enhancement in biomass after 20 d growing in standard solution, which contains a low concentration of total N (0.3 mg/L) and a high concentration of total P (15 mg/L) [3,41]. This agrees with a study showing that the concentration of nitrates in the medium didn’t affect phosphorus removal rates and the presence of NH_4_^+^-N showed no effect on the growth of azolla [4]. In fact the highest phosphorus uptake rate, specific growth rate and productivity of azolla in this research were observed in mineral media containing no added nitrogen [4].

**Figure 2:**
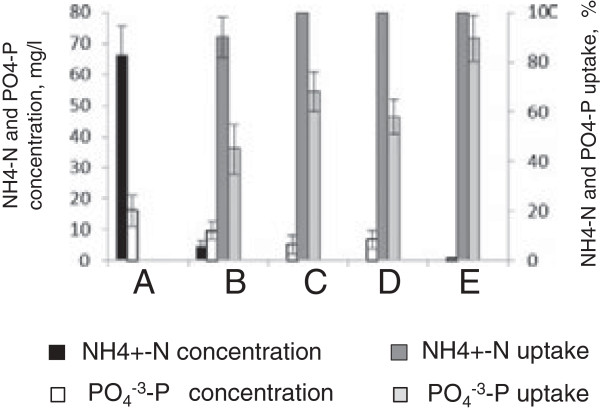
**Concentrations of NH**_**4**_^**+**^**-N and PO**_**4**_^**-3**^**-P and their uptake efficiency from 10**% **anaerobically digested swine wastewater (ADSW) by duckweed and azolla. (A)** Initial concentrations of NH_4_^+^-N and PO_4_^-3^-P in 10% ADSW. Nutrient concentrations and their uptake efficiency by: **(B)** duckweed at day 10; **(C)** duckweed at day15; **(D)** fresh duckweed at day 15; **(E)** fresh azolla at day 15.

To compare how duckweed and azolla could sequentially treat ADSW, we conducted three experiments in which (i) after 10 days of growing in 10% ADSW, duckweed was left to grow for another 5 days; or wastewater after day 10 was filtered (1-mm sieve) to remove duckweed and (ii) fresh duckweed fronds were added to the medium and left to grow for another 5 days; and (iii) azolla plants were added and also left to grow for 5 more days. Growing old and fresh duckweed for the next 5 days resulted in the complete removal of NH_4_^+^-N from medium but left 5.2 mg/L (68% removal) and 6.8 mg/L (58% removal) of PO_4_^-3^-P, respectively (Figure 2C and 2D). Lower absorption rates of fresh culture can be explained by the lag period required for acclimation of duckweed to the new environment [16]. The addition of fresh azolla removed NH_4_-N completely and significantly reduced PO_4_^-3^-P to 0.7 mg/L (96% removal) (Figure 2E). This suggests that duckweed and azolla can advantageously complement each other if sequentially used for recovery of N and P nutrients from the wastewaters.

The presence of microalgae and other organisms in wastewater has been shown to result in additional removal of nutrients [3,4,45]. As the surface of the water was not fully covered by duckweed and azolla plants in most of the experiments, light penetration resulted in the detectable growth of phototrophic algal populations (Figure 1D, G, Tables 1 and 2). Concentration of chlorophyll was used to estimate algal growth rates. Coverage of the water surface by duckweed reduced the concentration of chlorophyll in media from 6.1 μg/ml to 2.1 μg/ml. Denser coverage of the water surface by azolla after day 10 in 5 to 1.2% ADSW caused a near elimination of algal growth, as indicated by a reduction from 2.1 μg/ml to 0.1 μg/ml.

#### Seasonal variations in efficiency of nutrient uptake from synthetic swine wastewaters by duckweed and azolla

**Figure 3:**
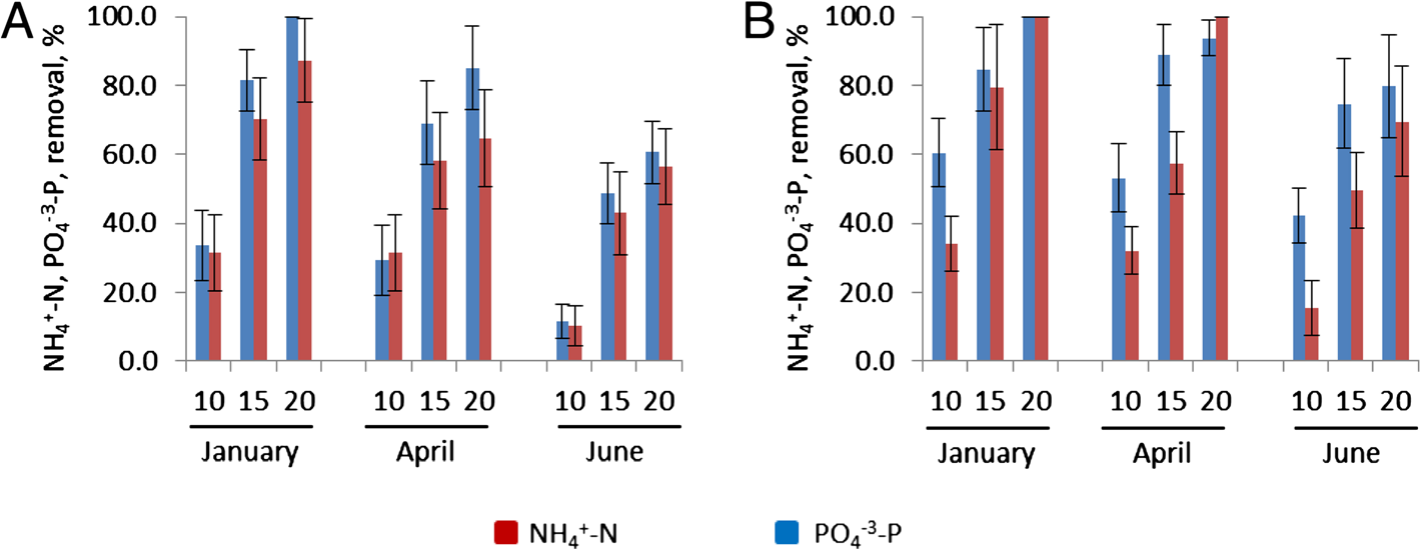
**Seasonal changes in efficiencies of nutrients removals from synthetic wastewater by duckweed and azolla. (A)** Duckweed; **(B)** azolla; 10, 15, 20 are the number of days from the start.

The growth rates of both azolla and duckweed were influenced by seasonal changes in temperature and solar radiation. Highest growth rates for both species were observed in experiments conducted in April, at 3.5 g dw/m^2^-day and 4.4 g dw/m^2^-day, respectively (Tables 3 and 4, Figure 3, Additional file 3). Lower growth and nutrient uptake rates were observed in June when growth of both plants was affected by low temperature as well as low solar irradiation (Additional file 3), with average plant density and growth rates of 45.8 g dw/m^2^ (2.3 g dw/m^2^/day) for duckweed and 67 g dw/m^2^ (3.3 g dw/m^2^/day) for azolla, correlating with reduced levels of N and P uptakes. During January when the water temperature increased occasionally up to +35°C (day) and 27°C (night) both duckweed and azolla showed low growth rates, 2.1 g dw/m^2^/day and 2.9 g dw/m^2^/day, respectively. Relatively high NH_4_^+^-N and PO_4_^-3^-P uptakes in both tanks were likely caused by an algal bloom. Growth of the mixed vegetation (plants and algae) was associated with the complete removal of NH_4_^+^-N from both tanks by day 20 and orthophosphate concentrations were also almost completely reduced, or completely reduced, over this period (Table 4, Figure 3, Additional file 3). An annual average growth rate of duckweed in synthetic wastewater under the climatic conditions of Melbourne, Australia was 2.6 g dw/m^2^-day (8.3 t/ha-yr). This growth rate is lower than those published by Cheng *et al*., 2002 [17], Cheng and Stomp, 2009 [38], and by Xu and Shen, 2011 [20], and is close to the growth rate of *Spirodella* spp. growing outdoor in mineral nutrient medium (3.1 g dw/m^2^-day reported by Reddy and Debusk, 1985 [25]). The annual average uptake of NH_4_^+^-N and PO_4_^-3^-P were 10.2 mg/l-day or 2.5 g/m^2^-day (9.1 t/ha-year) and 1.0 mg/L-day or 0.24 g/m^2^-day (0.8 t/ha-year), respectively. The NH_4_^+^-N and PO_4_^-3^-P uptake rates were lower than rates obtained for remediation of swine wastewater by Mohedano *et al*., 2012 a,b [9,10] but similar to rates published by Xu and Shen, 2011 [20], Cheng *et al*., 2002 a,b [16,17] and were higher than rates observed by Sims and Hu, 2013 [39].

**Table 3:** Seasonal changes in growth rates of duckweed, nutrients removals and chlorophyll concentration in synthetic wastewater

**Months**	**Days from start**	**Growth density, g dw/m2**	**Growth rate, g dw/m2-day**	**NH4-N initial, mg/l**	**NH4-N final, mg/l**	**NH4-N uptake rate, mg/l-day**	**PO4-P initial, mg/l**	**PO4-P final, mg/L**	**PO4-P uptake rate, mg/l-day**	**N/P uptake**	**Chl A + B, μg/ml**
**January**	10	NA	NA	250.0	166.1 ± 18	8.4 ± 4	30.0	20.7 ± 6	0.93 ± 0.14	9	45.7 ± 12
	15	NA	NA		46.2 ± 8	13.5 ± 6		8.9 ± 4	1.4 ± 0.9	9.6	19.3 ± 8
	20	42.5 ± 10	2.1 ± 0.9		0.0	12.5 ± 7		3.9 ± 2	1.3 ± 0.7	9.5	12.3 ± 6
**April**	10	NA	NA	250.0	176.7 ± 29	7.3 ± 3	30.0	20.6 ± 8	0.9 ± 0.4	7.8	15.8 ± 5
	15	NA	NA		77.1 ± 11	11.5 ± 3		12.6 ± 6	1.20 ± 0.9	9.9	7.5 ± 3
	20	71.1 ± 16	3.5 ± 0.9		37.1 ± 7	10.6 ± 4		10.6 ± 3.0	0.9 ± 0.4	10.9	2.5 ± 1.2
**June**	10	NA	NA	250.0	221.1 ± 30	2.8 ± 2	30.0	26.9 ± 11	0.31 ± 0.2	9.3	5.8 ± 2
	15	NA	NA		128.1 ± 28	8.1 ± 4		17.1 ± 8	0.86 ± 0.2	9.4	4.9 ± 2
	20	45.8 ± 9	2.3 ± 0.7		98.1 ± 11	7.5 ± 3		13.1 ± 6	0.845 ± 0.2	8.9	2.9 ± 1

N, nitrogen; P, phosphorus; NA (not analyzed).

**Table 4:** Seasonal changes in growth rates of azolla, nutrient removals and chlorophyll concentration in synthetic wastewater

**Months**	**Days from start**	**Growth density, g dw/m2**	**Growth rate, g dw/m2-day**	**NH4-N initial, mg/l**	**NH4-N final, mg/l**	**NH4-N uptake rate, mg/l-day**	**PO4-P initial, mg/l**	**PO4-P final, mg/L**	**PO4-P uptake rate, mg/l-day**	**N/P uptake**	**Chl A + B, μg/ml**
**January**	10	NA	NA	40.0	16 ± 6	2.4 ± 0.8	20.0	13.2 ± 4	0.68 ± 0.4	3.5	33.7 ± 10
	15	NA	NA		6.1 ± 3	2.3 ± 0.7		4.1 ± 2	1.06 ± 0.5	2.1	12.3 ± 8
	20	58.2 ± 8	2.9 ± 0.9		0.0	2.0 ± 0.9		0.0	1.0 ± 0.6	2	2.3 ± 0.5
**April**	10	NA	NA	40.0	18.7 ± 8	2.1 ± 1.0	20.0	13.6 ± 4	0.64 ± 0.3	3.3	12.8 ± 4.8
	15	NA	NA		4.5 ± 2	2.4 ± 0.7		8.5 ± 1	0.76 ± 0.3	3.1	0.5 ± 0.2
	20	88.5 ± 18	4.4 ± 0.7		2.5 ± 1.1	1.9 ± 0.9		0.0	1.0 ± 0.6	1.9	0.0
**June**	10	NA	NA	40.0	23.1 ± 6	1.7 ± 1.0	20.0	16.9 ± 5	0.31 ± 0.1	5.5	8.8 ± 1.8
	15	NA	NA		10.1 ± 4	2.0 ± 1.1		10.1 ± 5	0.7 ± 0.2	3	3.9 ± 1.2
	20	67.2 ± 6	3.3 ± 0.9		8.1 ± 3	1.5 ± 0.8		6.1 ± 3	0.7 ± 0.3	2.3	2.7 ± 1.2

N, nitrogen; P, phosphorus; NA (not analyzed).

The annual average yield of azolla grown in synthetic wastewater was 3.7 g dw/m^2^-day (13.5 t/ha-yr). This value is lower than that published by Song *et al*., 2012 [41], (11.25 g/m^2^-day) growing in mineral media and fish farm wastewater (6.25 g/m^2^-day) [30]. The growth rate was similar to that reported by Costa *et al*., 2009 [4] (3.4-3.9 g/m^2^-day) and higher than those obtained for azolla growing outdoors in mineral nutrient medium (2.9 g dw/m^2^-day) [43]. The annual average uptake of NH_4_^+^-N and PO_4_^-3^-P by azolla was 1.8 mg/L-day or 0.4 g/m^2^-day (1.4 kg/ha-year) and 0.9 mg/l-day or 0.21 g/m^2^-day ( 0.7 t/ha-year), respectively. The NH_4_^+^-N and PO_4_^-3^-P uptake rates are similar to those reported by Song *et al*., 2012 [41] (2.5 mg/L-day) and (0.7 mg/L-day), respectively and by Costa *et al*., 2009 [4] (3 mg/L-day) and (0.5 mg/L-day), respectively.

### Biochemical composition of duckweed and azolla fronds

**Figure 4:**
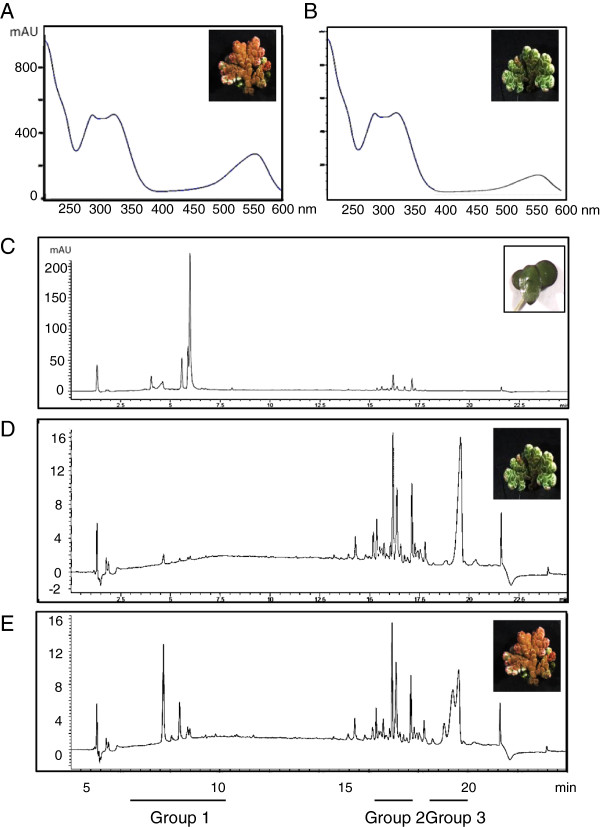
**Biochemical composition of duckweed and azolla fronds. (A)** and **(B)** Ultraviolet-visible spectrum between 200 nm and 600 nm of extracts from Azolla^red^ and Azolla^green^ fronds, respectively; **(C-E)** liquid chromatography-mass spectroscopy analysis of the molecules absorbing at the 520 nm range in duckweed, Azolla^green^ and Azolla^red^, respectively. Three groups of characterized molecules are described in Additional file 5.

Both, duckweed and azolla fronds contain visual red coloration suggesting accumulation of anthocyanin pigments in their tissues. Duckweed *L. punctata* contains red pigments on the lower, abaxial side of the fronds (Additional file 1), which are constantly present and do not reflect the cell response to abiotic stresses. Coloration of azolla fronds is dependent on their exposure to environmental stresses. Plants growing under outdoor conditions during cold winter or hot summer days showed accumulation of intense red pigments, mainly on the upper, adaxial sides of the fronds (Additional file 1). Ultraviolet/visual (UV/VIS) spectra between 200 nm and 600 nm of extracts from azolla fronds showed a specific peak between 500 nm and 600 nm representing a family of anthocyanins (Figures 4A, Additional file 4). Much smaller peaks were observed in plants with green fronds (Azolla^green^) compared to plants with red fronds (Azolla^red^) (Figure 4B). Liquid chromatography-mass spectroscopy (LC-MS) was used to analyze the composition of the molecules absorbing at the 520 nm range. Figure 4C-E shows three groups of characterized molecules. The first group of molecules eluting between 3 and 8 minutes had UV/VIS and MS characteristics consistent with regioisomers of glycosylated forms of pelargonidins, cyanidins or delphinidins (Additional file 5). Most of these molecules have been detected in plant species with colored fronds (Azolla^red^ and duckweed). Secondly, the later eluting groups of metabolites, (eluted between 15 and 17 minutes) belong to non-polar, less-stable molecules, which can easily be fragmented. It is likely that these metabolites are polyenes with varying levels of unsaturation and oxygenation. These molecules mainly exist in azolla samples. The third group of metabolites (eluted between 18 and 20 minutes) was detectable only in azolla samples, and had broad peaks in the UV/VIS chromatogram. These are believed to be lipophilic molecules, which do not ionize sufficiently well in electrospray ionization (ESI) to confidently assign a mass.

### Thermochemical conversion of duckweed, azolla and microalgae

Biomass from the three main vegetations, duckweed, azolla and algal populations, involved in recycling of nutrients in tanks 1 and 2 were collected and used as feedstocks for pyrolysis experiments. These three components of wastewater treatment represent very distant aquatic species: duckweeds are monocotyledonous plants, azolla are ferns and microalgae belong to unicellular photosynthetic algae. Biochemically these species contain different levels and compositions of essential metabolites, including proteins, starch and crude fat, as well as cel-wall components, which include cellulose, hemicellulose and lignin [3,16]. Among these species lignin was detected only in azolla and at much lower concentrations than in terrestrial plants [3]. It is important to know whether the differences in the biochemistry of these species could affect the composition of the three main pyrolysis products, bio-gas, bio-solids (char/ash) and bio-oil. It is also valuable to assess whether stress-induced differences in accumulation of secondary metabolites, for example, anthocyanins and flavonoids (Additional file 5) within the same azolla species (Azolla^red^ and Azolla^green^) can affect the composition of pyrolysis products. For this series of experiments azolla biomass was separated into plants with red and green fronds prior to pyrolysis experiments.

### Thermogravimetric analyses of duckweed, azolla and microalgae

Investigation of the thermal behavior of duckweed, azolla and microalgae at a wide range of temperatures (25 to 950°C) was carried out using a thermogravimetric analyzer (TGA). The results of the proximate analysis of the samples, including, the content of primary and secondary volatiles, fixed (solid) carbon and ash (that is, inorganic components of the samples) are summarized in Additional file 6. Total volatiles collected at 120 to 950°C represent 61 to 71% of total product, with the highest level observed in algae, which also contain the highest proportion of primary volatiles collected at 120 to 650°C. The plant species (duckweed and azolla) showed a slightly reduced proportion of fixed carbon (18 to 19% versus 20% in algae), but much greater amounts of ash (7.3 to 9.8% versus 4.1% in algae).

**Figure 5:**
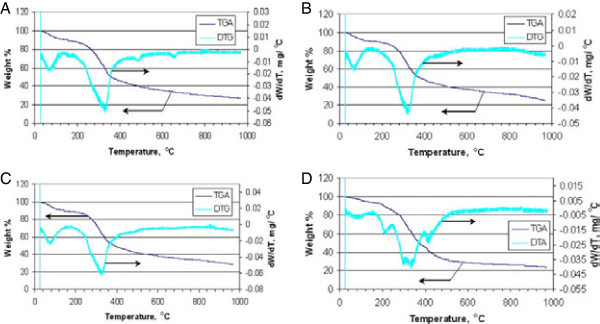
**Thermogravimetric/derivative thermogravimetric patterns of biomass species. (A)** Duckweed; **(B)** azolla^green^; **(C)** azolla^red^; **(D)** microalgae. TGA, thermogravimetric analysis; DTG: derivative thermogravimetric analysis.

Thermogravimetric analyses of the pre-dried samples of duckweed, azolla and microalgae at a temperature range of 25 to 950°C and heating rate of 20°C/minute are depicted in Figure 5. In general, the results of the analyses are consistent with the complex structure of aquatic biomass, comprising several classes of natural compounds: proteins, lipids, and carbohydrates, each of which is characterized by a distinct thermal signature. TGA/ derivative thermogravimetric (DTG) analysis-patterns of all analyzed samples show three distinctive stages of thermal degradation. The first stage, occurring at temperatures up to 160 to 180°C, is a dehydration stage, where water molecules adsorbed on the samples’ surface as well as those bound within the inner cells are released. All the samples tested exhibited a similar pattern in the first stage of thermal degradation. The second stage of thermal decomposition of all tested biomass samples occurs in the temperature range between 200°C and 400°C, where the majority of volatile compounds are released, with maximum release occurring at 320 to 330°C. During this stage, the chemical bonds of different bio-polymers start breaking, with less thermally stable compounds decomposing first. As a consequence, the TGA/DTG profiles of the tested samples showed some dissimilarity. The intensive peak (320 to 330°C) in the DTG curve of all samples can be attributed to hemicelluloses [46,47]. The cellulose decomposition peak, which typically manifests itself in the temperature range of 350 to 370°C [46,47] is probably overlapped with that of hemicelluloses. A similar profile for the green macroalgae *Codium fragile* has been reported by Daneshvar *et al*., 2012 [48], where hemicelluloses thermally degraded faster than cellulose, with the latter breaking down at between 250 and 407°C, or higher [48].

A distinct shoulder in the DTG profiles of Azolla^red^ in the lower-temperature region (270 to 290°C) is probably due to protein. This implies that protein content of these samples is probably higher than that of duckweed and Azolla^green^, which do not show these shoulders. It has been reported [49] that the DTG curve of a marine microalga shows an intensive peak at 285°C (at the same heating rate of 20°C/minute, as in our analyses), which was assigned to microalgae protein. The degradation of proteins in macroalgae (*Ulva lactuce*) samples in the temperature range of 280 to 340°C was shown by Trinh *et al*., 2012 [50]. Relatively low thermal stability of proteins has also been emphasized by other authors [51].

It can be seen that the DTG thermogram of algae is drastically different from that of both duckweeds and azolla, in that it exhibits two peaks in the low and high temperature ranges of the second stage. The peak in the low temperature range (200 to 210°C) could result from degradation and evaporation of oily compounds as well as thermally unstable functional groups of various macromolecules. This observation agrees with the reported finding that prior to decomposition of hemicellulose macromolecules in macroalgae, oily compounds degraded and evaporated from the sample at about 170 to 230°C [48]. The DTG peak at 400 to 410°C in all likelihood relates to decomposition of hydrocarbon chains of fatty acid components of the major lipid constituents. Kebelman *et al*., 2013 [52] conducted TGA analysis of lipid components extracted from the green microalga *Chlorella vulgaris* and found that the DTG of lipids exhibited a major decomposition peak at 400°C. The third stage of thermal degradation occurs at temperatures above 450°C. Weak peaks at about 470°C in the DTG of duckweed and both azolla samples may indicate the presence of small amounts of lignin-like compounds. Thermal degradation of lignin in macroalgae [50] and plants [46,50,53] shows peaks in this temperature range. DTG of algae does not show the presence of a similar (lignin) peak. Peaks at very high temperatures (above 600°C) are typically attributed to carbonization of char and decomposition and volatilization of inorganic compounds (ash) [48].

### Pyrolysis of duckweed, azolla and microalgae

#### Distribution of pyrolysis products

**Figure 6:**
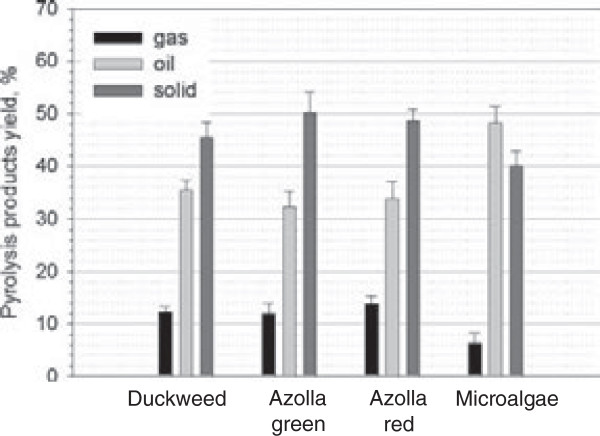
Production of bio-oil, bio-gas and bio-char after pyrolysis of duckweed, azolla and microalgae.

The major biomass pyrolysis products: gas, liquid and solids (char) and their distribution are shown in Additional file 7 and Figure 6, respectively. All experiments showed rather good mass balances (greater than 93%). Plant samples showed an enhanced proportion of gas and solid fractions compared to microalgae. On average, pyrolysis of plant samples generated 13% gas, 49% solid and 33% liquid fractions. No significant differences were observed in the proportion of gas and solid fractions between Azolla^red^ and Azolla^green^ plants. Algae produced higher yields of the liquid fraction (47.3%) with a correspondingly lower proportion of gas (6.3%) and solid (39.9%) fractions. Among duckweed, azolla and microalgae, only pyrolysis of microalgae has previously been intensively studied and reported [32]. The yields of liquid products were within the range of 20 to 50% dw, with the optimum temperature for production generally in the range of 300 to 450°C. Reactions at temperature above 500°C tended to give more gaseous products.

### Analysis of the products of duckweed, azolla and microalgae pyrolysis

#### Analysis of gaseous products of pyrolysis

Additional file 8 shows the results of gas chromatography (GC) analysis of gaseous products of pyrolysis, consisting mainly of CO and CO_2_ and small amounts of H_2_ and light (C1 to C4) hydrocarbons: methane, ethane, ethene, propane, propene and butanes/butenes. CO_2_ is the predominant component of the pyrolysis gas in all samples, with similar proportions observed in duckweed and azolla (average 77%) and algae (81%). Lower percentages of CO were found in algae (7% versus an average of 18% in duckweed and azolla). Azolla^red^ showed a slightly higher proportion of all gaseous components (except CO) than Azolla^green^ plants. Microalgae showed the higher proportion of gaseous hydrocarbons, methane (C1), ethane/ethene (C2), propane/propane (C3) and butane/iso-butane/sec-butanol/tert-butanol (C4) compared to duckweed and azolla.

#### Analysis of bio-liquid products of pyrolysis

**Figure 7:**
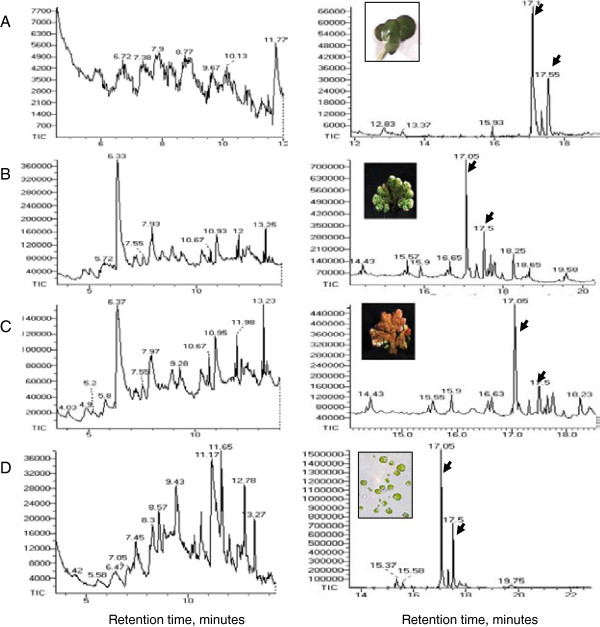
**Gas chromatography-mass spectroscopy chromatograms of bio-oil from duckweed, azolla and microalgae. (A)** duckweed; **(B)** azolla^green^; **(C)** azolla^red^; **(D)** microalgae. Arrows show two peaks, at 17.05 minutes and 17.55 minutes, representing a phytol molecule.

Bio-oils produced by pyrolysis of duckweed, azolla and microalgae are dark-brown viscous liquids (Additional file 7). GC-MS chromatograms of dichloromethane-dissolved bio-oil samples and retention times of the peaks of individual components of bio-oils are shown in Figure 7. The peak assignments and the list of molecules identified by the search-match feature of the MS software are summarized in Table 5. Only those peaks with a high degree of certainty, over 90%, are included in this list. In general, all analyzed samples produced a mixture of low molecular weight compounds representing different derivatives of aromatic and non-aromatic heterocyclic compounds, and oxygenated compounds, such as phenols, toluene, pyrrole, indolizine, indole, piperidine, pyran, cyclopentene and cyclohexene. Most of these molecules were previously found in the liquid products of pyrolysis of micro- and macro-algae [32,54]. Phytol, 3,7,11,15-tetramethyl-2-hexadecen-1-ol, acyclic diterpene alcohol, the product of the degradation of chlorophyll is the most abundant pyrolysis product and was detected in all analyzed samples (Table 5, Additional file 4): 3,7,11,15-tetramethyl-2-hexadecene (15.5- to 15.93-minute retention time), a derivative of phytol was also detected in all samples, but at lower concentrations. Most of the bio-liquid products of the duckweed *L. punctata* were previously identified in the pyrolysis products of another duckweed, *L. minor*[35,36]. Characteristically, the liquid composition of both of these duckweed species showed no hydrocarbons other than phytol. Interestingly, no phytol or other hydrocarbons were detected after pyrolysis of some representatives of seaweed, brown algae *Laminaria hyperborea* and *Fucus vesticulosus*, [54].

**Table 5:** Gas chromatography-mass spectroscopy analysis of bio-oil products of duckweed, azolla and microalgae pyrolysis

**Retention time, minutes**	**Duckweed**	**Azolla (green)**	**Azolla (red)**	**Microalgae**
4.3			Toluene	Toluene
4.9			3-Methyl furan and/or 3-Cyclopenten-1,2 diol	
5.2			Tetrahydro-4H-pyran-4-ol	
5.5			4-Cyclohexene-1,2-diol	
5.58				2-Methyl-1H-Pyrrole
5.72		2,4,5-Trimethyl 1H-imidazole		
5.8			1,2-Diemthyl-2-pyrroline	
6.18				Piperidine
6.33		Phenol	Phenol	Phenol
6.72	2,3-Dimethyl-1H-Pyrrole			2,3-Dimethyl-1H-Pyrrole
6.83				4-Ethyl-2-methyl-1H- pyrrole
7.38	2-Methyl phenol	2-Methyl phenol	2-Methyl phenol	
7.9	3-Methyl phenol	3-Methyl phenol	3-Methyl phenol	
8.3				3-Ethyl-2,5-dimethyl-1H-pyrrole
8.38				3-Ethyl-2,4-dimethyl-1H-pyrrole
8.77	4-Methyl phenol			
9.28			Dodecane	
9.33				2-Isopropylpyrazine
9.43				3-Ethyl-2,4,5-Trimethyl-1H-Pyrrole
9.67	4-Ethyl phenol			
10.13	2-Ethyl phenol			
10.67		Tridecane	Tridecane	
10.83				Indole
11.77	Indolizine	Indolizine	Indolizine	
11.98		Tetradecane	Tetradecane	Tetradecane
12.83	3-Methyl Indolizine			3-Methyl Indolizine
13.23		Pentadecane	Pentadecane	Pentadecane
13.37	4-Methyl 1H-indole			
14.43		Hexadecane	Hexadecane	Hexadecane
15.28				6,9-Heptadecadiene
15.37				1,15-Pentadecanediol
15.38				8-Heptadecane
15.57		Heptadecane	Heptadecane	Heptadecane
15.93	3,7,11,15-Tetramethyl-2-hexadecene	3,7,11,15-Tetramethyl-2-hexadecene	3,7,11,15-Tetramethyl-2-hexadecene	3,7,11,15-Tetramethyl-2-hexadecene
16.65		Octadecane	Octadecane	
17.1 to 17.55	Isomers of 3,7,11,15-Tetramethyl-2-hexadecen-1-ol (phytol)	Various isomers of 3,7,11,15-Tetramethyl-2-hexadecen-1-ol (phytol)	Various isomers of 3,7,11,15-Tetramethyl-2-hexadecen-1-ol (phytol)	Various isomers of 3,7,11,15-Tetramethyl-2-hexadecen-1-ol (phytol)
17.8				Nonadecane
18.25		Palmitoleic acid	Palmitoleic acid	
18.65		Eicosane (C_20_)		
19.58		Heneicosane (C_21_)		
19.75				Linolenic acid

Unlike duckweed, the bio-liquid fraction of pyrolysis products from azolla and microalgal samples showed the presence of diesel-range long-chain saturated alkanes (paraffins) sizing from C_12_ to C_21_, dodecane, tridecane, tetradecane, pentadecane, hexadecane, heptadecane, octadecane, nonadecane, eicosane and heneicosane (Table 5). Fatty acids were also found in bio-liquid products of azolla and algae: omega-7 monounsaturated palmitoleic (9-hexadecenoic) acid was found in azolla and omega-3, unsaturated linolenic fatty acid was found in algae-derived pyrolysis samples. Pyrolysis bio-oil produced from other algal strains contained a similar mixture of long-chain lipid-derived alkanes [32,55-59]. These types of molecules were not detected in pyrolysis products of duckweed (this research and [35]).

Accumulation of anthocyanins containing three phenolic rings (Additional file 4) in Azolla^red^ samples was associated with the presence of the low molecular weight, five- and six-membered heterocyclic, non-aromatic rings in the liquid pyrolysis product. These metabolites are derivatives of pyrroline, cyclopenten, pyrans, cyclohexene and pyrroline and were missing in Azolla^green^ products. In contrast, pyrolysis products of Azolla^green^ contained higher alkanes, eicosane (C_20_) and heneicosane (C_21_), which were absent in Azolla^red^.

Currently, phytol, a major product identified in all analyzed samples, is commercially used as a precursor for the manufacture of synthetic forms of vitamin E [60] and vitamin K1 [61]. Phytol is also used in the fragrance industry and in cosmetics, shampoos, toilet soaps, household cleaners and detergents [62]. Its worldwide use has been estimated to be approximately 0.1 to 1.0 metric tons per year (http://www.ifraorg.org/view_document.aspx?docId=22594). Phytol is being explored by Argonne National Laboratory (USA) for the production of drop-in biofuels (http://www.transportation.anl.gov/engines/multi_dim_model_biofuels.html). The physical and chemical properties of phytol such as density, cetane number and heat of combustion are close to that of diesel fuel. C_12_-C_21_ straight-chain alkanes found in the pyrolysis products of azolla and algae can be recovered from bio-oil and added directly to diesel fuels.

#### Analysis of solid products of pyrolysis (bio-char and ash)

As well as petrochemicals, biomass represents a resource for the production of other value-added bio-solids such as bio-char and ash (which is the inorganic component of bio-char remaining after its combustion). The soil-amending properties of bio-char (also known as charcoal) are well-known. Reported studies demonstrated that adding charcoal to soil substantially increased seed germination, plant growth and crop yields (the latter could be increased by up to 200%) [63]. In particular, charcoal applied to soil increases its nutrient- and moisture-holding capacity, supports microbial communities and activates the root activity, thus promoting biomass growth; these effects are attributed to higher exchange capacity, changes in pH and surface area, and other factors [63]. Biomass ashes carry many of the alkali elements (Li, Na, K, Mg, Ca, et cetera) and some transition elements (Fe, Mn, Cu, et cetera), which makes them a good candidate for use as fertilizers [64].

Bio-char has traditionally been used for the production of activated carbons (AC) and other carbonaceous products [65]. Because of its availability, stability and low cost AC can be used as adsorbents, catalyst supports, or utilized directly as catalysts, replacing conventional expensive catalysts such as noble metals, Pt, Rh, Ir or Ni that can be easily poisoned by aggressive substances such as sulfur, phosphorus and nitrogen compounds [56]. We have previously shown that CO_2_-treated bio-char exhibits appreciable initial catalytic activity in bio-gas reforming with production of syngas [36].

**Figure 8:**
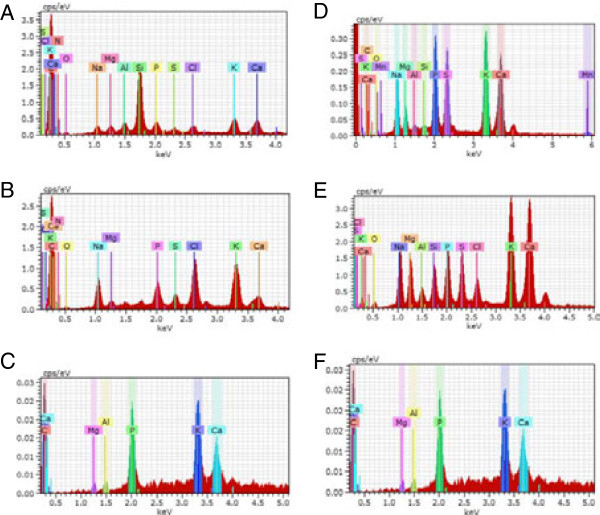
**Energy-dispersive x-ray spectroscopy analysis of bio-char and ash from duckweed, azolla and microalgae. (A-C)** Bio-char; **(D-F)** ash; **(A, D)** duckweed, **(B, E)** azolla and **(C, F)** microalgae.

The yield of bio-char was higher for azolla (average 49%) and duckweed samples (45%), compared to microalgal samples (39%) (Figure 6). Unlike the composition of bio-oil fraction, which varied greatly between duckweed and azolla*,* the composition of bio-char products, analyzed by energy-dispersive x-ray spectroscopy (EDX) showed similar features for duckweed, azolla and microalgae. The detected elements include: C, K, Na, Cl, Ca, P, S, O, Mg, Si, Al (where, naturally, carbon was observed as the most abundant element in all analyzed bio-char samples) (Figure 8, Additional file 9). Following C, K showed next highest abundance in all analyzed samples (5 to 7 wt% and 2 to 3 at%, (weight% and atomic%, respectively) respectively). The next most abundant elements for duckweed were, in descending order: Ca, P and Na; for azolla: Na, Cl and Ca, and for algae: P, O and Ca. Scanning electron microscopy images of bio-chars from duckweed, azolla and algae are shown in Additional file 10.

Based on the thermogravimetric patterns obtained in a flowing-air atmosphere, the ash content of duckweed and azolla were estimated at a weight fraction of 7.3% and 8.5%, respectively, which far exceeds that of terrestrial plant biomass (typically less than weight fraction of 2% [36]). Unlike the composition of bio-char products, the components of inorganic ash produced by the combustion of plant samples contain very little carbon. The chemical composition revealed a set of elements similar to those detected in bio-char, occurring in more or less similar ratios, with Ca representing the most abundant element (21 to 25 wt% and 14 to 17 at% (weight% and atomic%, respectively)) (Figure 8, Additional file 11). Si and P were also abundant elements in duckweed. A comparison of scanning electron micrographs (SEM) of bio-char and ash indicated that the basic structural features of samples remained intact and were not affected by thermolysis (Additional file 10).

## Conclusion

The Human Development Report of the United Nations Development Programme (UNDP) (http://hdr.undp.org/en/content/human-development-report-2006) has focused on the global water crisis as one of the most serious problems facing humanity today. The demand for fresh water doubles every 10 to 15 years, as a result of rising domestic consumption and the increasing needs of industry. Phytoremediation strategies based on the abilities of certain plants and microalgal populations to recycle nutrients from wastewaters offer attractive solutions for water pollution problems. Aquatic plants, aggressively colonizing wetlands are widely used for phytoremediation and represent attractive feedstock for biofuel production. A dual application of aquatic plants for wastewater treatment and production of value-added chemicals offers an ecologically friendly and cost-effective solution for water pollution problems and renewable energy production.

In our study, representatives of Australian aquatic floating macrophytes, *L. punctata* and *A. filiculoides*, were studied for bio-filtration and recycling of swine wastewater from the main contaminants: phosphates, ammonia and microalgal vegetations, and as feedstock for conversion of generated biomass into a number of value products such as bio-gas, bio-solids and bio-liquid. The main conclusions from this research include the fact that duckweed and azolla, which efficiently absorb N and P components of wastewater, can advantageously complement each other if sequentially used for efficient recovery of N and P nutrients from a swine wastewater. Another advantage of the application of azolla for bioremediation relates to its ability to efficiently inhibit the growth of microalgal and cyanobacterial populations, by producing a thick mat on the surface of the water, which prevents penetration of oxygen and light. The presence of microalgal populations in final water-effluents is highly undesirable and in many cases requires additional purification stages.

Analysis of pyrolysis products showed that azolla and algae produce a similar spectrum of bio-oils, which in turn are very different from the products obtained from duckweed samples. This large spectrum of petrochemicals and straight-chain C_10_-C_21_ alkanes can be directly used as diesel fuel supplement, or as a glycerine-free component of biodiesel. Pyrolysis of duckweed produces precursor bio-oil components, which can be used as feedstocks for so-called green gasoline and diesel biofuel production using different existing techniques, such as catalytic hydrodeoxygenation. The presence of N- and S-containing compounds in bio-oils (for example, indol, piperidine, et cetera) may require an additional step of hydrodenitrification (HDN) and hydrodesulfurization (HDS) in order to avoid catalyst poisoning during the upgrading of bio-oils into finished fuels (HDN and HDS processes are commonly practiced at refineries).

Biochemical analysis of the pyrolysis products of azolla plants showed that changes in profiles of secondary metabolites, such as flavonoids and anthocyanins, triggered by redirection of intermediate molecules within the phenylpropanoid pathway in response to environmental stresses [66,67], result in modifications in the composition of pyrolysis products. This suggests that targeted alterations to the composition of pyrolysis products may be feasible.

## Methods

### Growing duckweed and azolla

The *L. punctata* was acquired from Robert Bell, Bio-Tech Waste Management Pty Ltd and *A. filiculoides* was collected from the lake in the Melbourne Royal Botanic Garden, Melbourne, Australia. The swine lagoon wastewater was provided by Dr J Hill, Termes Consulting Ltd, Melbourne. The swine wastewater was treated anaerobically. The concentrations of NH_4_^+-^N and PO_4_^-3^-P in the ADSW were 680.7 mg/L and 145.7 mg/L, respectively. Concentration of other inorganic nitrogen in the wastewater, such as NO_3_^-^-N was very low and not reported. The initial pH of ADSW was 8.6, which is too high to grow healthy plants. The laboratory scale experiments on nutrient strength were carried out in 250-mL plastic cups filled with 200 mL ADSW of a specific concentration: 100.0%, 50.0%, 25.0%, 15.0%, 10.0%, 5.0%, 2.5%, or 1.5%. Concentrations of NH_4_^+^-N and PO_4_^-3^-P in these dilutions are shown in Additional file 2. The media were sterilized by autoclaving for 20 minutes at 121°C. The plastic cups were placed in a 23°C growth chamber with a 16-h photoperiod and a photosynthetic photon flux density of 50 μm/m^2^-s provided by three fluorescent tubes [17]. Five replicates were included for each treatment. Destructive sampling was conducted to evaluate the nutrient tolerance of duckweed and to monitor the nutrient level and duckweed growth at day 10. The medium and duckweed in each box were mixed every day. To reduce algal growth the cups were covered at the sides with black sheets allowing light penetration only from the top.

Seasonal variations in duckweed and azolla growth and nutrient recovery from the synthetic swine wastewater were analyzed from April 2012 to January 2013 in Melbourne, Australia with samples collected in April, (mid-autumn) with average day/night temperatures of 26°C and 15°C respectively, June, (early winter), the coolest month with average day/night temperatures of 14°C and 7°C, respectively, with occasional frosty mornings reaching 2 to 5°C and January, (mid-summer), the hottest month, with average day/night temperatures of 26°C and 15°C, respectively, with occasional days reaching 30 to 35°C (http://www.bom.gov.au/climate/averages/tables/cw_086071.shtml). At the beginning of the experiments duckweed (tank 1) and azolla (tank 2) covered approximately 30% of the surface of the water (Additional file 3). Wastewater treatment tanks, each having a dimension of 1.3 m (length) × 0.7 m (width) × 0.50 m (depth) were filled with 250 L of synthetic swine wastewater which was formulated to closely resemble the nutrient profile, total ionic strength, pH, and buffering capacity of swine lagoon effluent [15]. The concentration of NH_4_^+^-N was 250 mg/L for duckweed and 40 mg/L for azolla, while the concentration of PO_4_^-3^P was 30 mg/L for duckweed and 20 mg/L for azolla. Three replicates were conducted for each treatment. Specific growth rate and maximum plant density (considering biomass at the end of the assays and the area of tanks) were evaluated according to Rai *et al*., 2006 [68]. Productivities were calculated based on plant densities and specific growth rates for each time interval. Medium samples were analyzed for ammonia cations, nitrate and phosphate anions and pH. Concentrations of cations and anions were measured using an ion chromatography system Dionex ICS-1100 (Thermo Scientific, USA). The dry weights were analyzed immediately after sampling drying samples at 80°C overnight.

### Analysis of chlorophyll concentrations

Algal biomass was collected by centrifugation. Chlorophyll was extracted with ethanol and extinction at 649, 665, and 750 nm was determined. Chlorophyll concentration (Chl, μg/mL) was calculated using the equation [69]:

$$\mathrm{Chl}=\left(6.1\;\left(E665–E750\right)+20.04\left(E649–E750\right)\right)K$$

where *E* is extinction at the corresponding wavelength, K is the dilution factor, and 6.1 and 20.04 are extinction coefficients.

### Pyrolysis experiments

Pyrolysis experiments were carried out in a quartz tube reactor with a diameter of 12 mm. The biomass samples (2.0 g) were dried at 110°C overnight before experimental runs. Heating of the quartz tube reactor was carried out using a tube furnace which was controlled by a temperature controller (Omega CSC32, Omega Engineering, Inc., USA). Two thermocouples (one external to the tube and one internal) were used to monitor pyrolysis temperature. According to the experimental design, the furnace (and the quartz reactor) was vertically aligned, so that the liquid products dripped into the condenser assembly which was chilled using ice. The pyrolysis reaction was carried out at a temperature of 500°C and the flow rate of Ar sweep-gas of 100 mL/minute at atmospheric pressure; the duration of the experiments was 15 minutes. After condensation of the liquid product, the gas passed through a glass-wool filter before being collected in a gas-sampling bag. The condenser was weighed before and after the reaction to obtain the weight of the liquid product (bio-oil) collected. The solid product bio-char was dislodged from the pyrolysis reactor after the experiment and weighed. The volume of the pyrolysis gas was measured and the gas sample was analyzed by GC. Three replicates were conducted for each treatment. Similar slow pyrolysis conditions applied for pyrolysis of macro- and microalgal biomass have been reported in the literature [70]. In order to increase the yield of bio-oil in large-scale production units, fast pyrolysis conditions will need to be applied.

### Thermogravimetric analysis

TGA and DTG analysis of the duckweed and azolla samples were performed using the PerkinElmer Diamond TG/DTA instrument. The samples were used as is, without drying. The samples were loaded into the instrument and heated from 50 to 950°C at a heating rate of 20°C/minute. TG-pyrolysis (using He as carrier gas) was used to determine the moisture content, volatiles and fixed carbon. TG-combustion (using air as carrier gas) was used to determine the ash content. The loss of weight between 25 and 120°C was used to calculate the moisture content of the sample. The volatile content of the samples was determined from the loss of weight between 120 and 650°C (primary volatiles) and 650 and 950°C (secondary volatiles). The ash content of the sample was determined from the amount of solids remaining at the end of the combustion run. Fixed carbon was calculated by subtracting the ash content from the solids remaining at the end of the pyrolysis run.

### Analysis of bio-oil

The liquid product of pyrolysis (bio-oil) was analyzed by GC-MS. The sample of bio-oil was dissolved in aliquot amounts of dichloromethane (DCM) and injected into the Agilent 6890 N (Agilent Technologies, Santa Clara, CA, USA) coupled to a JEOL GCMate-II (JEOL Ltd., Tokyo, Japan) GC-MS. Typical GC-MS parameters used in the analyses were as follows: helium as carrier gas at a flow rate of 2 mL/minute, column: HP-5 ms (60 m × 0.32 mm × 0.25 μm), injection port temperature: 300°C, GC-MS interface temperature: 250°C, a sample injection volume: 10 μL, split ratio: 50:1.

### GC analysis of gaseous products of pyrolysis

Pyrolysis gas analysis was performed using a Varian 450 (Varian Inc., USA) GC with thermal conductivity detector (TCD) for permanent gases and flame ionization detector (FID) for hydrocarbon gases. Argon was used as carrier gas and three columns were used for separation-PLOT alumina/KCL, Molecular sieve 5A and Haysep Q.

### Analysis of bio-char and ash

The solid products of duckweed and azolla pyrolysis, bio-char and ash, were analyzed by EDX using the Hitachi TM-3000 (Hitachi High-Technologies Corporation, Japan) SEM with Bruker Quantax 70 (Bruker Nano, USA).

### LC-MS analysis

For LC-MS analysis, 100 mg of dry powder was extracted with 1 ml of methanol:water (80:20, v:v) with sonication for 5 minutes. After centrifugation the extract was transferred to a HPLC vial. Three replicates were conducted for each treatment. The extracts (5 μl injected) were analyzed using a 150 mm × 2.1 mm ThermoScientific 1.9-μm C18 HPLC column fitted to an Agilent series 1200 HPLC device (Agilent, Walbronn). Metabolites were eluted from the column using a gradient mobile phase, A (0.1% formic acid in water, Riedel-de Haen) and B (0.1% formic acid in acetonitrile, Riedel-de Haen) at 0.2 ml/minute. Initial conditions (98% A) then a linear gradient to 100% B over 15 minutes, was maintained for 10 minutes before returning to the initial gradient conditions. The compounds were detected with a Thermo Scientific LTQ Velos orbitrap mass spectrometer (Waltham, MA, USA; Thermo, Bremen, Germany), operating in the ESI mode with a HESI probe for positive data acquisition. Each extract was analyzed using an LTQ Velos Orbitrap (ThermoScientific) in ESI positive mode over a mass range of 80 to 2000 amu with data stored in profile mode. For structure elucidation, positive-mode accurate mass data and MS2 information (resolution set to 30,000) were obtained in a data-dependant fashion with dynamic exclusion on (repeat-count 3, repeat-duration 20 s).

### Statistical analysis

All treatments in this study were conducted in triplicate (except experiments with growing duckweed and azolla in wastewater, where five replicates were used for each treatment). The experimental data were subjected to the one-way analysis of variance (ANOVA) as implemented in the GraphPad InStat 3 statistics platform. The Tukey simultaneous test was conducted to determine the statistical differences between treatments. In order to ascertain that the observed variations in growth rates, efficiency of nutrients uptake and the yield of pyrolysis products were statistically significant, the probability (P) values were determined. A 95% confidence level (P ≤0.05) was applied for all analyses.

## Abbreviations

A. azollae: *Anabaena azollae* Strasburger; AC: activated carbons; A. filiculoides: *Azolla filiculoides*; azollagreen: azolla plants with green leaves; azollared: azolla plants with green leaves; ADSW: anaerobically digested swine wastewater; EDX: energy-dispersive x-ray spectroscopy; dw: dry weight; DTA: differential thermal analysis; DTG: derivative thermogravimetric analysis; FID: flame ionization detector; GC-MS: gas chromatography-mass spectroscopy; HDN: hydrodenitrification; HDS: hydrodesulfurization; HPLC: high-performance liquid chromatography; LC-MS: liquid chromatography-mass spectroscopy; L. punctata: *Landoltia punctata*; SEM: scanning electron micrograph; TCD: thermal conductivity detector; TGA: thermogravimetric analyses; UV/VIS: ultraviolet-visual.

## Competing interests

The authors declare that they have no competing interests.

## Authors’ contributions

AM designed the project and conducted the main part of the biotechnological experiments, was involved in interpretation of data for the work, made substantial contributions in writing a manuscript, and approved the final version for publication. NM made substantial contributions to designing and conducting the pyrolysis experiments, critically revising generated data, contributed to writing and final approval of the manuscript, to analysis and to interpretation of data, and critically revised and approved the final version for publication. AG made substantial contributions to designing and conducting the pyrolysis experiments, critically revising generated data, contributed to writing and final approval of the manuscript, to analysis and to interpretation of data, and critically revised and approved the final version for publication. ASB made substantial contributions to interpretation of data for the work and drafting the work, and approved the final version for publication. TS made substantial contributions to interpretation of data for the work and drafting the work, and approved the final version for publication. KK analyzed the nutrient composition of wastewater, made substantial contributions to interpretation of data, and contributed to writing and final approval of the manuscript. SR carried out the biochemical studies of duckweed and azolla, made substantial contributions to interpretation of data and statistical analysis, and contributed to writing and final approval of the manuscript. AFM was involved in growing plants and algae, analyzing their biomass and growth rates, contributing to the statistical analysis of generated data, and writing a manuscript and its final approval. MT was involved in growing plants and algae and analyzing their biomass and growth rates, and contributed to the statistical analysis of generated data, and writing a manuscript and its final approval. All authors were involved in final approval of the version to be published and agreed to be accountable for all aspects of the work. All authors read and approved the final manuscript.

## Supplementary Material

Additional file 1 **Images of three major components involved in swine wastewater treatment. (A)** Azolla fronds: green (left) and stressed red (right), adaxial sides, bar = 5 mm; **(B)** transverse section of azolla fronds, ×20 magnification, bar = 20 μM. Arrowheads are showing filamentous cyanobacteria *A. azollae* in cavities of azolla fronds, Lc, leaf cavity; **(C)** duckweed, *L. punctata* fronds: adaxial (left) and abaxial (right) sides, bar = 1 mm; **(D)** algal population; bar = 100 μM. Click here for file

Additional file 2 Initial nutrient concentrations and pH of different dilutions of anaerobically digested swine wastewater (ADSW). Click here for file

Additional file 3 **Duckweed and azolla growth in synthetic wastewater under outdoor conditions.(A)** Starting density, (30%) of duckweed (tank 1) and azolla (tank 2); **(B)** full coverage of water surface in both tanks in April 2012; **(C)** final coverage of tanks in June 2012; **(D)** algal growth in tank 1 in April at day 10 (left flask), day 20 (middle flask) and in tank 2 at day 20 under dense azolla coverage (right flask). Click here for file

Additional file 4 Chemical formulas of selected chemicals. Click here for file

Additional file 5 Biochemical analysis of flavonoids from duckweed and azolla by liquid chromatography-mass spectroscopy (LC-MS). Click here for file

Additional file 6 Proximate analysis of duckweed, azolla and microalgae. Click here for file

Additional file 7 Main pyrolysis products: (A) bio-oil, (B) bio-char and (C) ash. Click here for file

Additional file 8 Gas chromatography (GC) analysis of gaseous products of duckweed, azolla and microalgae samples (in % volume). Click here for file

Additional file 9 **Elemental analysis of bio-char from duckweed, azolla and microalgae.** Click here for file

Additional file 10 Scanning electron microscope (SEM) images of bio-char and ash from duckweed, azolla and microalgae. Click here for file

Additional file 11 Elemental analysis of ash from duckweed, azolla and microalgae. Click here for file
