# Supplementary figures and images for: Dual application of duckweed and azolla plants for wastewater treatment and renewable fuels and petrochemicals production
Source: Biotechnol Biofuels. 2014 Feb 28;7:30. doi: 10.1186/1754-6834-7-30 (PMC3944989; doi:10.1186/1754-6834-7-30)

## Slide 1
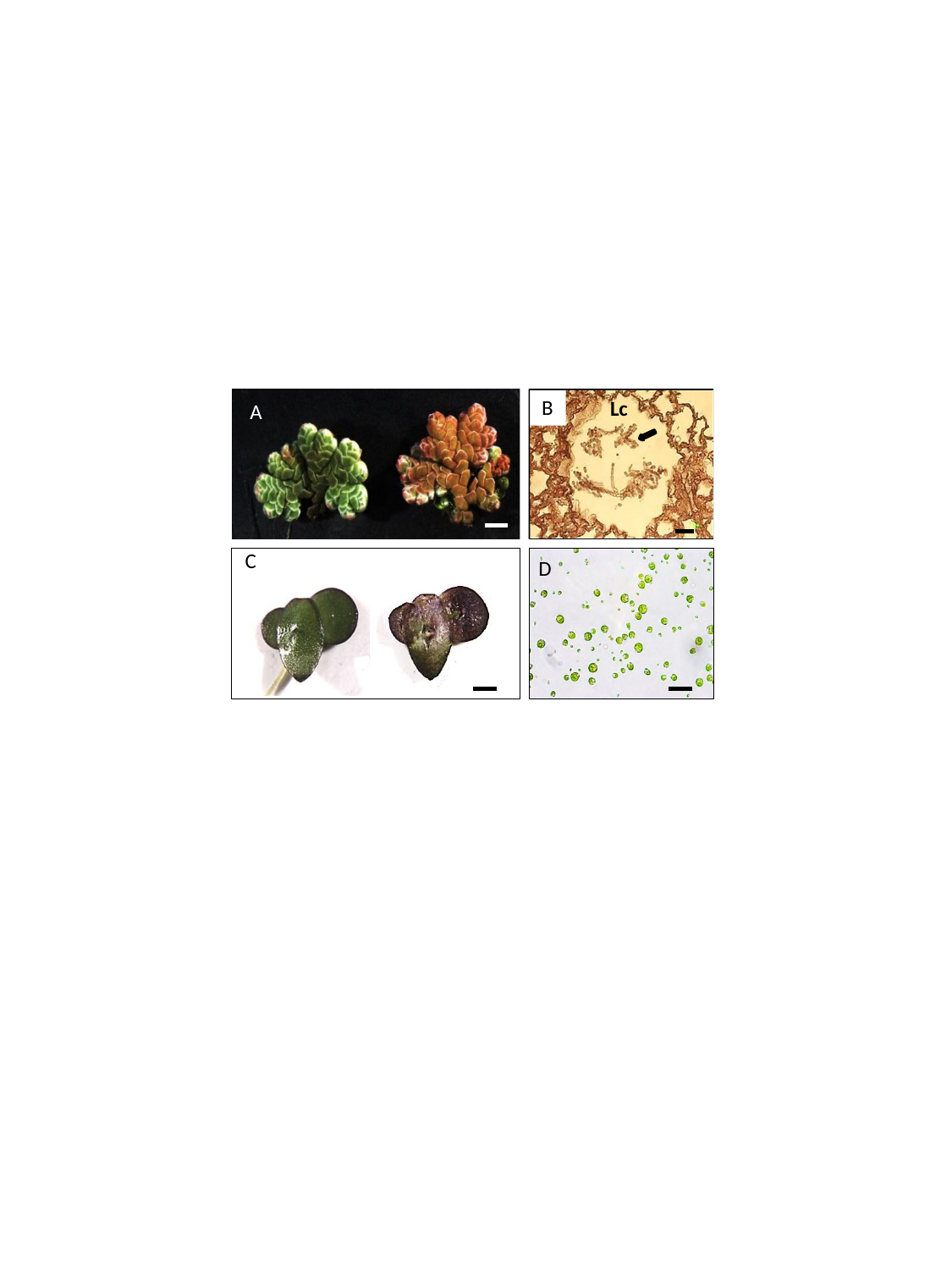

B
Lc
A
C
D

Supplement: Additional file 1 — Images of three major components involved in swine wastewater treatment. (A) Azolla fronds: green (left) and stressed red (right), adaxial sides, bar = 5 mm; (B) transverse section of azolla fronds, ×20 magnification, bar = 20 μM. Arrowheads are showing filamentous cyanobacteria A. azollae in cavities of azolla fronds, Lc, leaf cavity; (C) duckweed, L. punctata fronds: adaxial (left) and abaxial (right) sides, bar = 1 mm; (D) algal population; bar = 100 μM. [file 1754-6834-7-30-S1.pptx]

## Slide 1
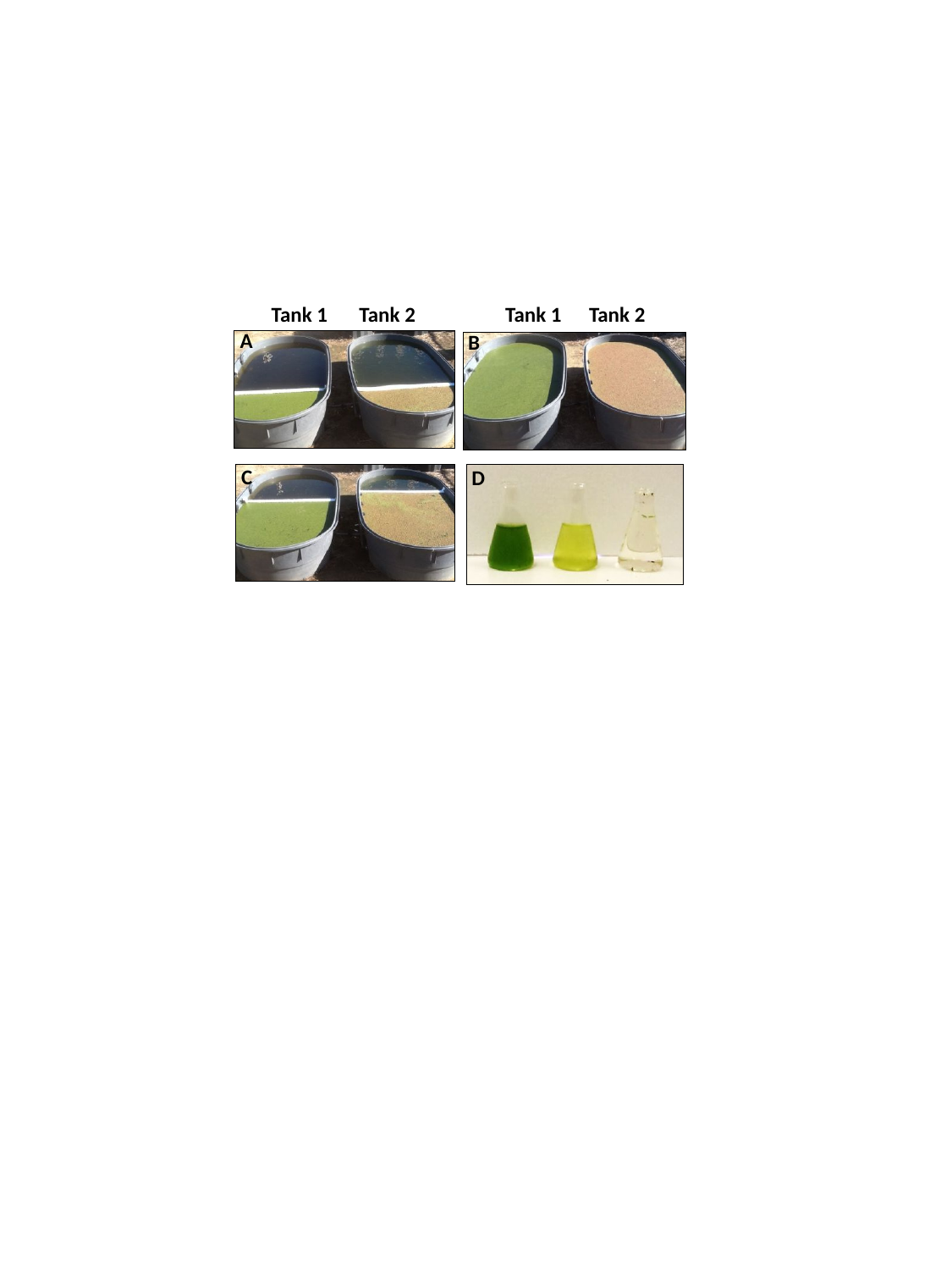

Tank 1
Tank 2
Tank 1
Tank 2
A
B
C
D

Supplement: Additional file 3 — Duckweed and azolla growth in synthetic wastewater under outdoor conditions.(A) Starting density, (30%) of duckweed (tank 1) and azolla (tank 2); (B) full coverage of water surface in both tanks in April 2012; (C) final coverage of tanks in June 2012; (D) algal growth in tank 1 in April at day 10 (left flask), day 20 (middle flask) and in tank 2 at day 20 under dense azolla coverage (right flask). [file 1754-6834-7-30-S3.pptx]

## Slide 1
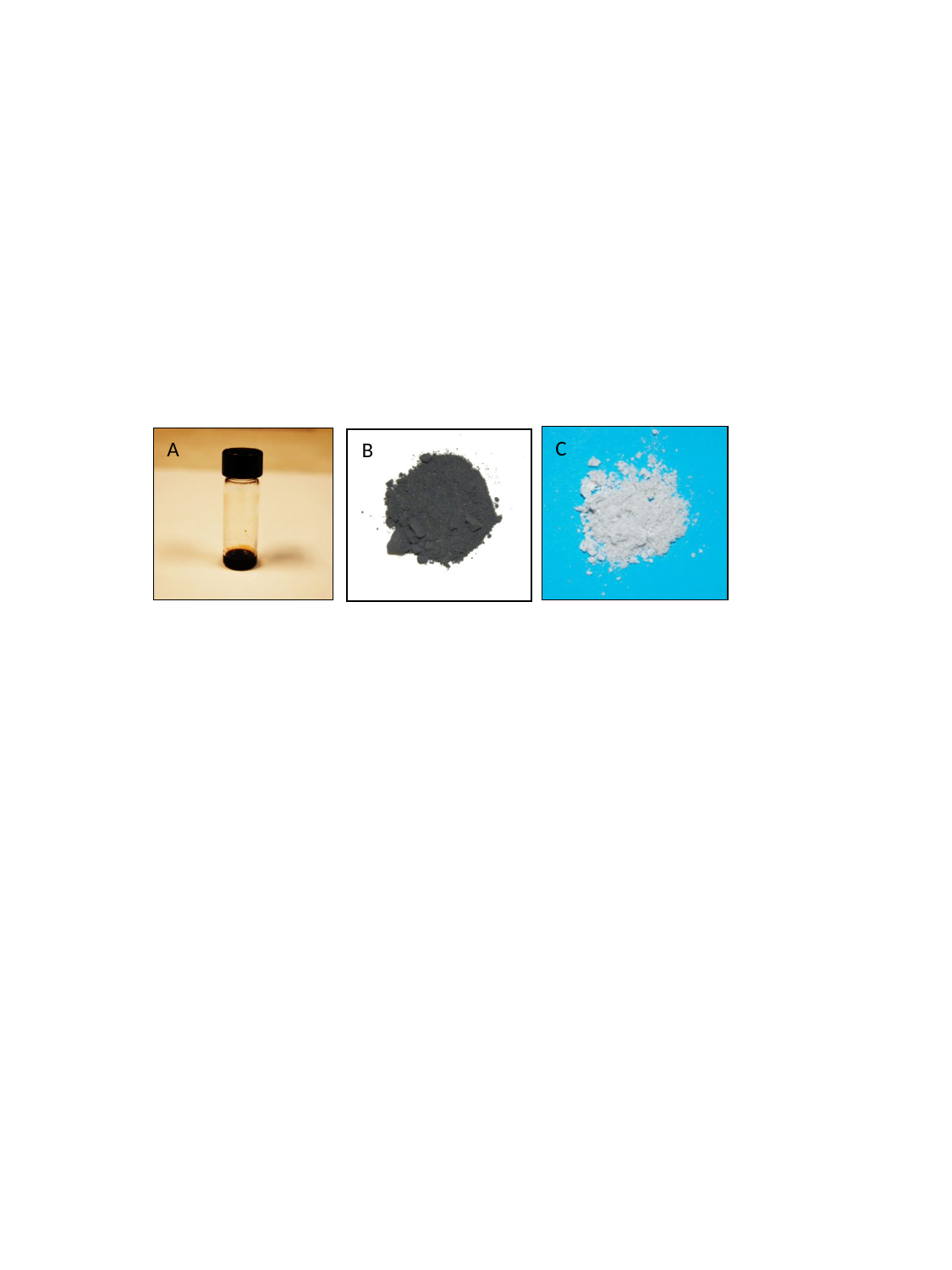

C
A
B

Supplement: Additional file 7 — Main pyrolysis products: (A) bio-oil, (B) bio-char and (C) ash. [file 1754-6834-7-30-S7.pptx]

## Slide 1
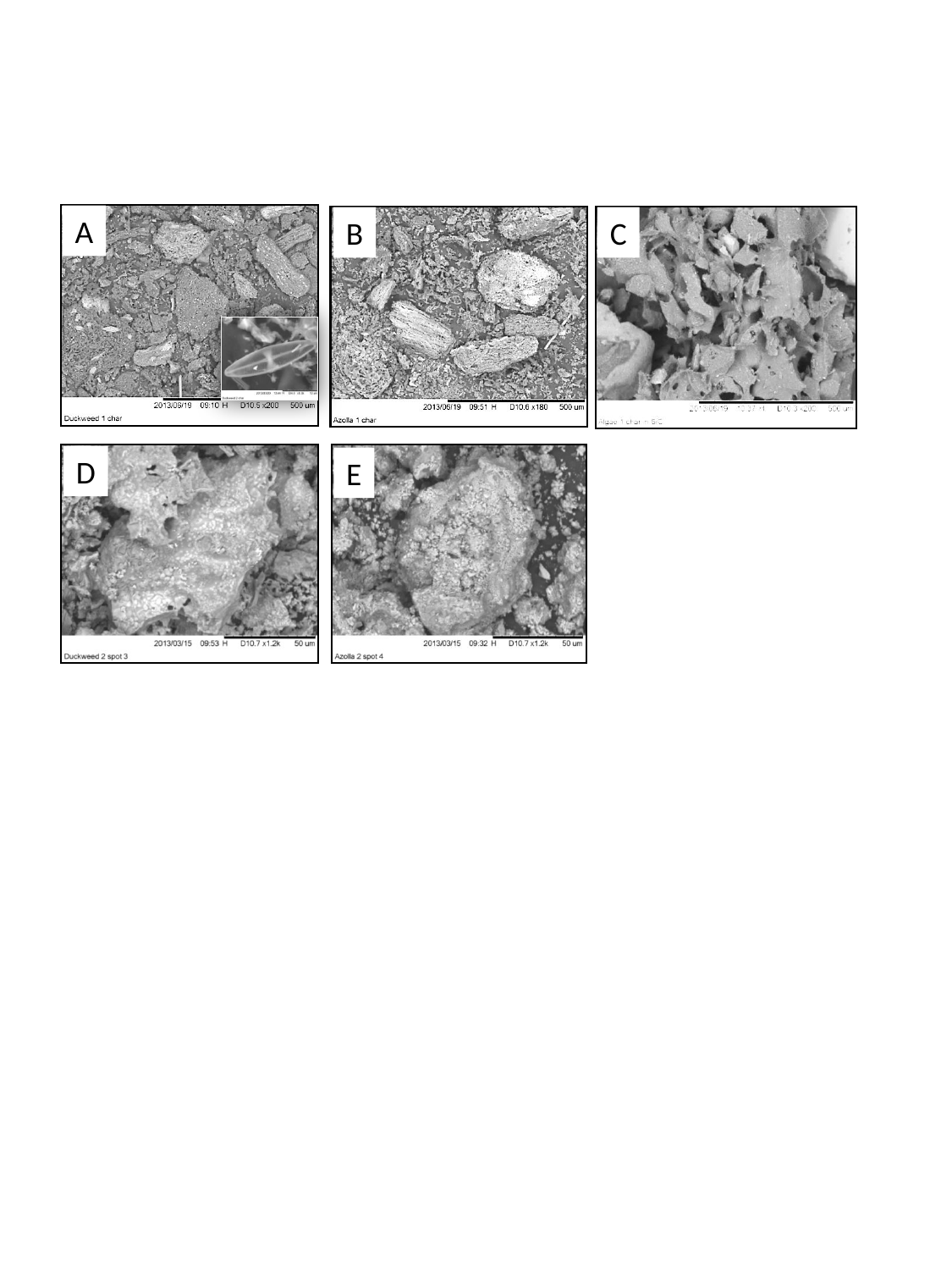

A
B
C
D
E

Supplement: Additional file 10 — Scanning electron microscope (SEM) images of bio-char and ash from duckweed, azolla and microalgae. [file 1754-6834-7-30-S10.pptx]
